# An Assessment of the Role of DNA Adenine Methyltransferase on Gene Expression Regulation in *E coli*

**DOI:** 10.1371/journal.pone.0000273

**Published:** 2007-03-07

**Authors:** Aswin Sai Narain Seshasayee

**Affiliations:** Genomics and Regulatory Systems Group, EMBL-European Bioinformatics Institute, Wellcome Trust Genome Campus, Cambridge, United Kingdom; University of the Western Cape, South Africa

## Abstract

N6-Adenine methylation is an important epigenetic signal, which regulates various processes, such as DNA replication and repair and transcription. In γ-proteobacteria, Dam is a stand-alone enzyme that methylates GATC sites, which are non-randomly distributed in the genome. Some of these overlap with transcription factor binding sites. This work describes a global computational analysis of a published Dam knockout microarray alongside other publicly available data to throw insights into the extent to which Dam regulates transcription by interfering with protein binding. The results indicate that DNA methylation by DAM may not globally affect gene transcription by physically blocking access of transcription factors to binding sites. Down-regulation of Dam during stationary phase correlates with the activity of TFs whose binding sites are enriched for GATC sites.

## Introduction

Dam is an N6-Adenine methyltransferase, which methylates GATC sites soon after replication. Methylation is a bacterial version of an immune response to phages. It has been described as a signal that influences DNA-protein interactions [1]. GATC sites have been shown to overlap with the binding sites of global transcriptional regulators, CRP and FNR, and thus influence their activity [2]. This would imply that a knockout of Dam should have drastic effects on gene expression.

This work analyses a recently published microarray data of a Dam mutant in order to assess the effect this has on transcription regulation. Further, GATC-containing TF binding sites are analysed in order to correlate any gene expression changes to Dam binding. Finally, a hypothesis concerning the balance between Dam binding and transcription regulation by the global factor CRP is presented.

## Methods

### Datasets

Microarray dataset for Dam mutant was obtained from Robbins-Manke et al [3]. One set of stationary phase microarray data was obtained from Tjaden et al. [4] and the other was downloaded from the ASAP database [5] in November 2005. FNR knockout microarray data on Affymetrix platform, which is used here as an example to assess the effect of the knockout of a global transcriptional regulator on gene expression, was obtained from Covert et al. and Kang et al. as raw data and processed as below [6], [7]. Literature derived datasets for (1) transcription factor binding sites (TFBS) (2) sigma factor binding sites/promoters (SFBS) and (3) transcription units were obtained from RegulonDB 5.0 [8]. COG functional category assignments for E coli were obtained from GenBank.

### Microarray data analysis

The raw CEL files were processed using the RMA procedure and differentially expressed genes were identified using LIMMA. RMA does not require a baseline array for normalization and is based on achieving quantile-quantile plots that are along the unit vector of the diagonal [9]. LIMMA uses a moderated t-test approach to identify differentially expressed genes [10]. For all microarray data except the *dam* mutant, differential expression was defined by a q-value of 0.05 following FDR multiple testing. For the *dam* mutant, the cutoff was 0.01 without multiple testing The reasoning is explained in context in the results section. All these calculations were carried out using Bioconductor [11].

### Functional category enrichment

Enrichment of specific functional categories among differentially expressed genes was carried out using the an F-test followed by FDR as used in FatiGO to identified enriched functional categories among differentially expressed genes [12]. This was done in R.

### Permutation tests

Permutation tests were used for certain analyses as described in the results. For this the pairings of TFs/Sigma factors to binding sequences were randomly shuffled around.

### Tetranucleotide profiling

Tetranucleotides in coding sequences and TFBS were counted using the compseq program in the EMBOSS package [13]. For the F-test, the FatiGO script implemented in R was used. An alternative scoring scheme was also used to test for enrichment. The formula for enrichment in this approach is:
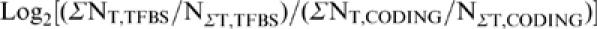
where N_T,TFBS_ is the number of occurrences of tetranucleotide T within TFBS, N_∑T,TFBS_ is the sum of the counts of all tetranucleotides within TFBS, ∑N_T,CODING_ is the number of occurrences of tetranucleotide T within coding regions and N_∑T,CODING_ is the sum of the counts of all tetranucleotides within coding regions. A two-fold enrichment would correspond to a score of∼0.7.

## Results

### Dam mutant does not result in global changes in transcription

In recent years, three different microarray studies have analysed gene expression changes in *dam* E coli [3], [14], [15]. The most recent of these [3], for which the raw data is available in GEO, describes an overall increase in expression of about 200 genes in *Dam* using Affymetrix GeneChip arrays. For the current study, this raw data was reanalyzed. The data was normalised using RMA as in the above study. However, instead of the ANOVA analysis used in the above study, the moderated t-test approach of LIMMA implemented in Bioconductor [11] was used to identify differentially expressed genes. Following p-value adjustment with multiple testing, it was found that none of the genes showed a statistically significant change in gene expression at an FDR of 0.05. This is very unlike an FNR–one of seven global regulators defined by Martinez-Antonio and Collado-Vides [16]-knockout under anaerobic conditions [6], [7], which on normalisation with RMA followed by detection of differential expression with LIMMA and multiple testing with FDR results in 340–360 differentially expressed genes. While such a multiple correction approach is effective in normalizing for dependencies across genes, it can lead to a loss of sensitivity [17]. Hence, a more conservative approach of a raw p-value cut-off of 0.01 and a log (base 2) change of 0.7 (2-fold change) was used on the *Dam* dataset. 109 genes were differentially expressed (Table S1). This included RecA and LexA confirming the activation of DNA repair mechanisms. An over-representation of genes involved in translation was also observed (F-test as used in FatiGO [12], FDR q-value: 10^−15^). This is as observed in the original study [3]. However, the present analysis shows that no other functional category is enriched. The lack of significance of differential expression after multiple-correction might imply that these changes are subtle. The gene expression changes may be restrained due to the activity of a relatively less characterized methyltransferase SmtA [18], [19]. It may also arise because of variation in the extent of double strand breaks in the population as reported [3].

### Dam binding sites and gene expression changes are not correlated

Yet another dataset that was used in this study is experimentally verified transcription factor binding site (TFBS) and promoter sequence (PS) data downloaded from RegulonDB 5.0 [8]. A list of experimentally verified transcription units was used in conjunction with the above data in order to identify promoters of genes that are differentially expressed, which also contain the GATC motif. The 109 differentially expressed genes fell in 65 different transcription units. Of these 38 had an experimentally verified sigma factor binding sequence (SFBS) and 25 had at least one known TFBS.

If the change in expression levels were due to altered TF binding to DNA in the *dam* strain, then we would expect to see an over-representation of GATC containing SFBS and TFBS in the list of differentially expressed transcription units. However, this was not the case (Tables S2 and S3). Only 3 differentially expressed TFBS and SFBS contained GATC sites. This was just random (Z-scores of-1.5 and 0.005 for SFBS and TFBS respectively; control: 1000 randomly shuffled gene-SFBS/TFBS pairs). In fact a lack of such correlation was reported by Robbins-Manke et al. as well [3]. This implies that any change in expression levels, despite being subtle, observed in the microarray data cannot be attributed to the direct influence of methylation on transcription factor or sigma factor binding to the DNA. These transcriptional changes might be in response to damage to the DNA that is caused by reduced methylation or due to hitherto unexplained indirect effects.

### Tetranucleotide profiling for GATC

In an earlier work, over-representation of specific words in gene-upstream regions was described as a measure of the regulatory potential of such motifs and validated using comparison with known structures of DNA-protein complexes [20]. Hence, as further test for the role of GATC as a regulatory motif at the transcriptional level, tetranucleotide profiling analysis as described in the above publication was carried out. Instead of an arbitrary cut-off, as was used in the former work, the F-test-FDR test was used to test for enrichment. Further, for this test, enrichment of tetranucleotides within the experimentally validated TFBS in comparison to the coding sequences was tested. It was found that out of 256 possible tetranucleotides, 91 were statistically enriched (q-value<0.05) within TFBS. This includes CTAG, which is the core binding sequence for trp [21] and the met [22] repressors and is known to be highly restrained in the genome [23]. However, despite the fact that over 35%of all tetranucleotides are enriched in TFBS, GATC is not, thus adding further to doubts on its role as a transcriptional regulatory motif. This is true even when an alternative approach to identifying enriched tetranucleotides is used. In this approach a score was computed for each tetranucleotide so that it would define whether the word is enriched within TFBS or not. At a two-fold enrichment cut-off, 53 tetranucleotides were obtained. This list was a subset of the list obtained using the F-test and thus GATC is found to be absent here as well. These lists are provided as tables S4A and S4B.

### Dam, CRP, Sigma38 and stationary phase

**Figure 1:**
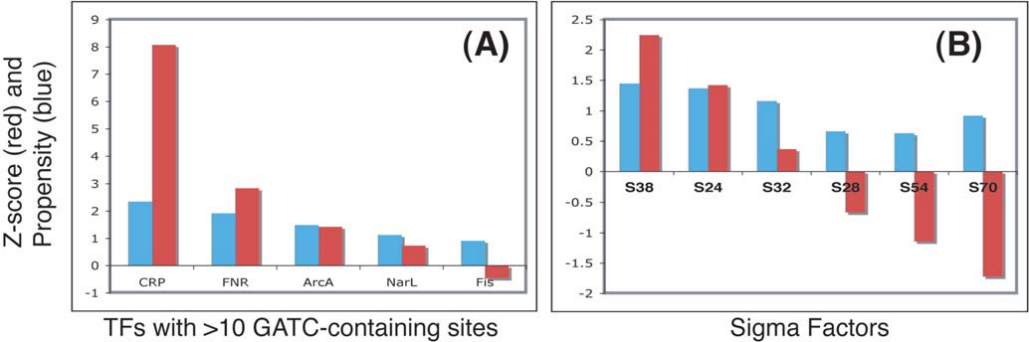
Plots showing the propensities and Z-score for various (A) transcription factor binding sites and (B) sigma factor binding sites to contain GATC.

In the next step of the study, TFs and Sigma factors whose binding sites were significantly enriched for GATC were identified (Fig. 1). Among TFs for which more than 10 sites contained a GATC sequence, only CRP and FNR showed significant enrichments (tested using 1000 shuffling of TF to site mapping). CRP (Z-score: 8) showed a much greater significance than FNR (Z-score: 2.5). In addition, a survey of Sigma factor binding sequences for GATC using the same permutation tests as used for TFBS shows that only the stationary phase/stress response sigma factor, Sigma 38 or rpoS, is enriched for GATC, though only slightly (Z-score: 2.5).

**Figure 2:**
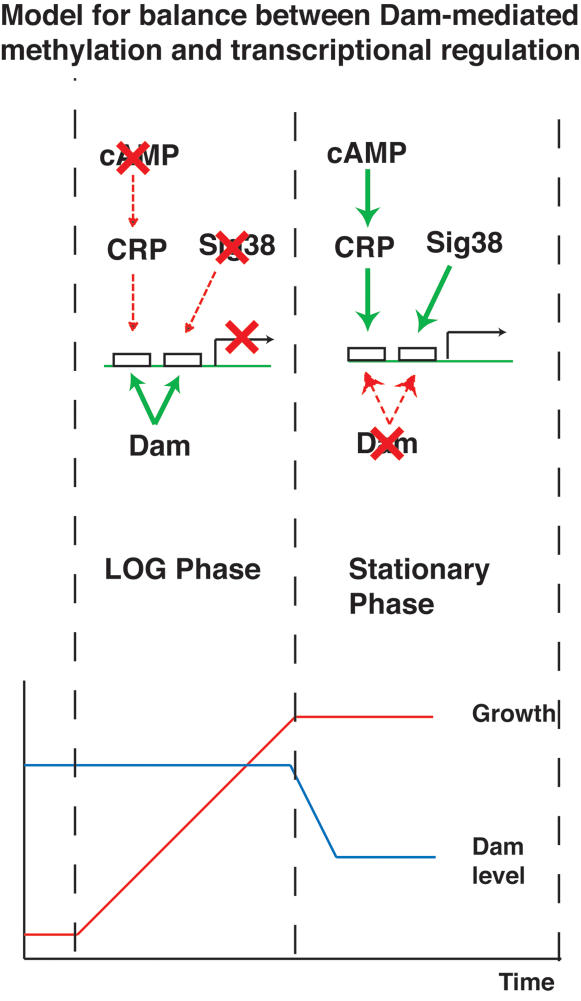
A schematic representation of the interplay between the growth phase, Dam-mediated methylation and transcriptional effects of CRP and Sigma38. This model is a hypothesis shows that Dam does not directly inhibit TF/Sigma binding and its downregulation in stationary phase correlates with the activation of CRP and Sigma38 whose binding sites which are enriched for GATC.

CRP, which is activated by cAMP signalling in response to glucose starvation, can be expected to be active during the stationary phase of growth in minimal medium and would therefore not be active under the conditions in which the above microarray data was obtained. Hence it is reasonable that CRP targets with GATC sites do not significantly change in expression levels in a Dam mutant grown in rich media. The same is applicable to SigmaS as well. However, the question is: how does CRP access its binding sites even during stationary phase when the sites are methylated? Two different publicly available Affymetrix microarray datasets for stationary phase E coli cells [4], [5] were mined (using comparisons with log phase arrays from the same experiment) for genes that were differentially expressed (moderated t-test from LIMMA and FDR<0.05) during the stationary phase (Table S5). It could be seen that Dam is consistently down regulated in the stationary phase (FDR of 10^−8^ and 10^−5^ in the two contrasts). This is consistent with results from a ten year old small-scale experiment showing that Dam levels are dependent on growth rate and that a ten-fold decrease in growth rate results in a four-fold fall in Dam levels [24]. This would result in reduced methylation during stationary phase, allowing CRP to bind its targets in newly divided cells. This could also be one reason why double strand breaks are induced during stationary phase [25]. This implies that Dam methylation does not really interfere with TF binding under conditions in which the TF might be expected to be active. Further the observation that only Sigma38 binding sites among those for all sigma factors show enrichment for GATC is further evidence to the above. This model is illustrated in Fig. 2.

### Conclusion

Despite the description of isolated cases where DNA methylation plays an important role in transcriptional regulation [1], it may not be a global player. Dam is down regulated in the stationary phase, which correlates with the enrichment of GATC in binding sites for CRP and Sigma 38, though the functional significance of the enrichment seen with FNR is not clear. [26]

## Supporting Information

Table S1 Genes differentially expressed in delta-dam mutant in comparison to wt. This is a reanalysis of data published by Robbins-Manke et al.
(0.01 MB TXT)

Table S2 Experimentally verified transcription units containing the genes that are differentially expressed in a dam mutant. Sequences shown are sigma factor binding sites
(0.00 MB TXT)

Table S3 Experimentally verified transcription units containing the genes that are differentially expressed in a dam mutant. Sequences shown are transcription factor binding sites
(0.00 MB TXT)

Table S4A Tetranucleotides enriched in TFBS against coding regions as seen from F-test FDR
(0.00 MB TXT)

Table S4B Tetranucleotides enriched in TFBS against coding regions as seen from propensity score
(0.00 MB TXT)

Table S5 Genes differentially expressed in two independent stationary phase vs. log phase contrasts
(0.05 MB TXT)
